# MIF Contributes to *Trypanosoma brucei* Associated Immunopathogenicity Development

**DOI:** 10.1371/journal.ppat.1004414

**Published:** 2014-09-25

**Authors:** Benoît Stijlemans, Lin Leng, Lea Brys, Amanda Sparkes, Liese Vansintjan, Guy Caljon, Geert Raes, Jan Van Den Abbeele, Jo A. Van Ginderachter, Alain Beschin, Richard Bucala, Patrick De Baetselier

**Affiliations:** 1 Department of Cellular and Molecular Immunology, Vrije Universiteit Brussel (VUB), Brussels, Belgium; 2 Myeloid Cell Immunology Laboratory, Vlaams Instituut voor Biotechnologie, Brussels, Belgium; 3 Department of Internal Medicine, Yale University School of Medicine, New Haven, Connecticut, United States of America; 4 Unit of Veterinary Protozoology, Department of Biomedical Sciences, Institute of Tropical Medicine, Antwerp, Belgium; Uniformed Services University of the Health Sciences, United States of America

## Abstract

African trypanosomiasis is a chronic debilitating disease affecting the health and economic well-being of many people in developing countries. The pathogenicity associated with this disease involves a persistent inflammatory response, whereby M1-type myeloid cells, including Ly6C^high^ inflammatory monocytes, are centrally implicated. A comparative gene analysis between trypanosusceptible and trypanotolerant animals identified MIF (macrophage migrating inhibitory factor) as an important pathogenic candidate molecule. Using MIF-deficient mice and anti-MIF antibody treated mice, we show that MIF mediates the pathogenic inflammatory immune response and increases the recruitment of inflammatory monocytes and neutrophils to contribute to liver injury in *Trypanosoma brucei* infected mice. Moreover, neutrophil-derived MIF contributed more significantly than monocyte-derived MIF to increased pathogenic liver TNF production and liver injury during trypanosome infection. MIF deficient animals also featured limited anemia, coinciding with increased iron bio-availability, improved erythropoiesis and reduced RBC clearance during the chronic phase of infection. Our data suggest that MIF promotes the most prominent pathological features of experimental trypanosome infections (i.e. anemia and liver injury), and prompt considering MIF as a novel target for treatment of trypanosomiasis-associated immunopathogenicity.

## Author Summary

Uncontrolled inflammation is a major contributor to pathogenicity development during many chronic parasitic infections, including African trypanosome infections. Hence, therapies should aim at re-establishing the balance between pro- and anti-inflammatory responses to reduce tissue damage. Our experiments uncovered that macrophage migration inhibitory factor (MIF) plays a pivotal role in trypanosomiasis-associated pathogenicity development. Hereby, MIF-deficient and neutralizing anti-MIF antibody-treated wild type (WT) *T. brucei*-infected mice exhibited decreased inflammatory responses, reduced liver damage and anemia (i.e. the most prominent pathogenicity features) compared to WT control mice. The reduced tissue damage coincided with reduced infiltration of pathogenic monocytic cells and neutrophils, whereby neutrophil-derived MIF contributed more significantly than monocyte-derived MIF to tissue damage. MIF also promoted anemia development by suppressing red blood cell production and enhancing their clearance. The clinical significance of these findings follows from human genetic data indicating that low-expression (protective) *MIF* alleles are enriched in Africans. The current findings therefore offer promise for human translation and open the possibility of assessing MIF levels or *MIF* genotype as an indication of an individual's risk for severe trypanosomiasis. Furthermore, given the unmet medical need of African trypanosomiasis affecting millions of people, these findings highlight MIF as a potential new therapeutic target for treatment of trypanosomiasis-associated pathogenicity.

## Introduction

African trypanosomiasis is a parasitic disease of medical and veterinary importance that adversely affects the public health and economic development of sub-Saharan Africa. The causative agents, trypanosomes transmitted by the tsetse fly (*Glossina spp*), are extracellular hemoflagellated protozoans that cause fatal diseases in mammals, commonly called sleeping sickness in humans (HAT, Human African Trypanosomiasis) or nagana in domestic livestock [1]. In the case of bovine trypanosomiasis, anemia is considered to be the most prominent pathogenicity feature and the major cause of death associated with the disease [2]. In fact, the main difference between trypanosusceptible and trypanotolerant animals relies in their capacity to control anemia development. The underlying mechanisms mediating trypanosome-associated anemia have been scrutinized in murine models [3]. The data collectively suggest that a strong pro-inflammatory/type I immune response, involving classically activated myeloid cells/macrophages (M1), is required for initial parasite growth control. Yet, if maintained, this response contributes to pathogenicity in general and anemia in particular in trypanosusceptible mice, resulting in reduced survival of the host. Hereby, myeloid cell hyperactivation was proposed to be involved in the extravascular destruction of red blood cells (RBCs) due to enhanced erythrophagocytosis by spleen and liver-associated M1 cells of the infected host [3], [4] causing trypanosome-associated anemia. Such type I immune response driven anemia resembles anemia of inflammation, also termed anemia of chronic disease (ACD), that is associated with chronic infections and sterile inflammations [5], [6].

Uncontrolled inflammation associated with persistence of M1 cells is also a major cause of liver injury and cachexia observed in trypanosusceptible animals, whereby the anti-inflammatory cytokine IL-10 was found to be detrimental to prevent these pathogenic features [7]. Hence, therapies should aim at re-establishing the balance between pro- and anti-inflammatory signals during the disease to avoid tissue damage. In this context, the glycosylphosphatidylinositol (GPI)-anchor of the Variant Surface Glycoprotein (VSG) coat was identified as a major parasite-derived molecule with a M1-activating potential [8]. Interestingly, a GPI-based treatment was found to protect against infection-associated cachexia, liver damage, anemia, and to prolong survival by modulating the myeloid cell activation state, *i.e.* forcing a transition from M1 to M2 (alternatively) activated myeloid cells during the course of infection [9].

The possibility to render trypanosusceptible animals more tolerant by modulating the activation state of myeloid cells offers an attractive model to identify genes and gene-products involved in the pathogenicity of African trypanosomiasis. In this context, a comparative gene expression analysis revealed that the macrophage migration inhibitory factor (MIF) expression was significantly reduced in mice rendered trypanotolerant upon GPI treatment. This “early response” cytokine is expressed by numerous cell types, including myeloid cells, and plays a key role in innate and adaptive immunity [10], [11]. MIF is a prominent inducer of systemic inflammation in many inflammatory diseases [12], [13]. It functions by recruiting myeloid cells to the site of inflammation [14], by inducing their differentiation towards M1 cells secreting TNF [15] and by suppressing p53-dependent apoptosis of inflammatory cells [16]. Since African trypanosomes trigger a persistent type I/M1 immune response in trypanosusceptible (e.g. *T. brucei brucei* (*T. brucei*)) infected mice, we evaluated the potential role of MIF in the development of infection-associated pathogenicity. More specifically, the effect of MIF on the infiltration of myeloid cells, liver damage and anemia development was investigated.

## Results

### 1. MIF expression levels show two distinct waves during *T. brucei* infection

**Figure 1:**
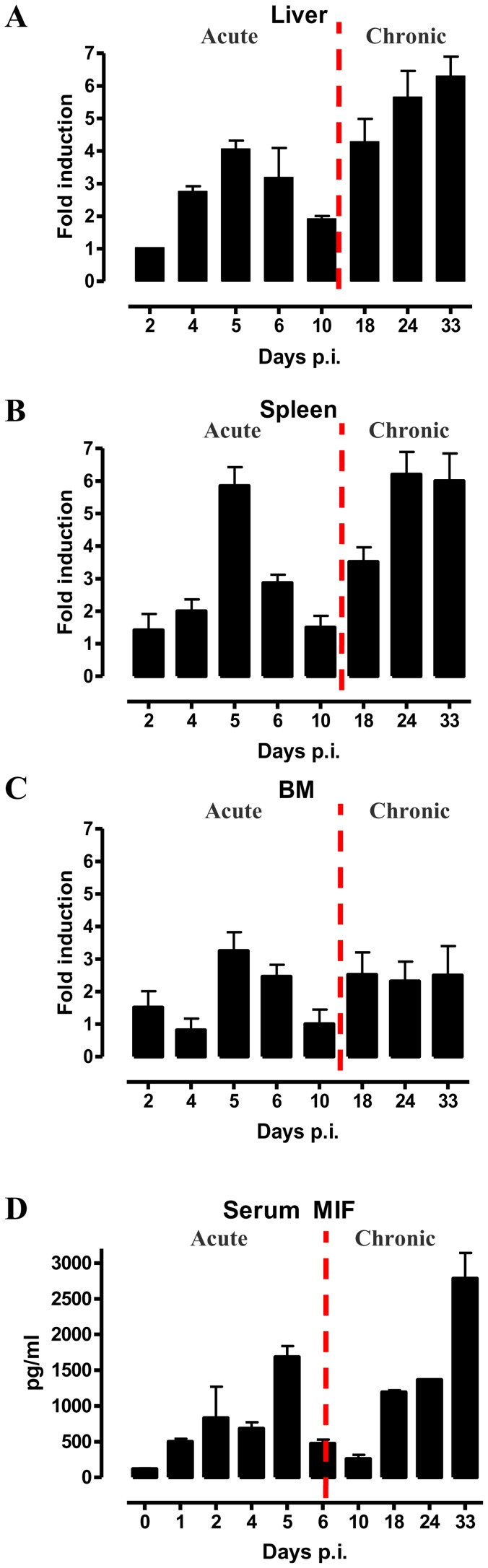
MIF expression exhibits biphasic profiles during *T. brucei* infection. *Mif* gene expression levels in the liver (A), spleen (B), bone marrow (BM) (C) and serum MIF protein levels (D) during infection in C57Bl/6 mice. The dashed line delineates the transition from the acute to the chronic phase of infection. Gene expression levels were normalized against *s12* and expressed relative to expression levels in non-infected mice. Results are representative for 2–3 independent experiments and presented as mean of 2–3 individual mice ± SEM.

As a first step towards evaluating the potential role of MIF during the course of *T. brucei* infection, we analysed its gene expression in different organs. As shown in Fig. 1A-C, MIF gene expression level in liver, spleen and bone marrow was characterized by two distinct phases, i.e. an initial increase during the acute phase of infection that returns back to the level of non-infected mice, followed by a second more progressive increase during the chronic phase of infection. Serum MIF protein levels followed the same kinetic as in the tested organs (Fig. 1D).

### 2. MIF deficiency correlates with reduced type I inflammation during *T. brucei* infection

**Figure 2:**
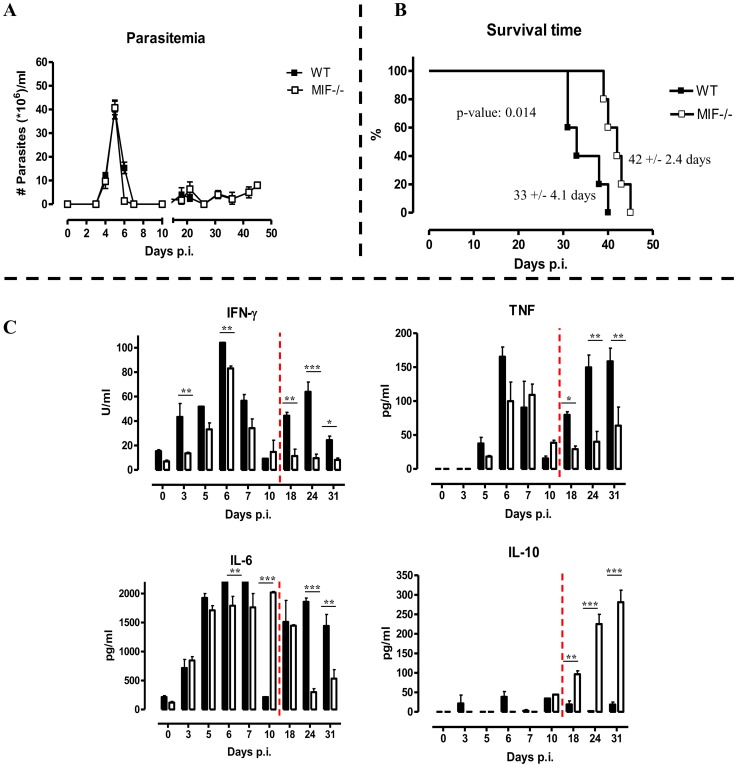
MIF deficiency confers protection and reduces inflammatory immune responses during *T. brucei* infection. Parasitemia (A) and survival (B) during infection in C57Bl/6 (WT, black box; *Mif* ^−/−^, white box) Mice. Results are representative of 2–5 independent experiments and expressed as mean of 5 individual mice ± SEM. (C) Serum cytokine levels of IFN-γ (upper left panel) (1 U = 100 pg), TNF (upper right panel), IL-6 (lower left panel) and IL-10 (lower right panel) in *Mif* ^−/−^ (white bars) and WT (black bars) mice. The dashed line delineates the transition from the acute to the chronic phase of infection. Results are representative of 3 independent experiments and presented as mean of 3 individual mice ± SEM (*: p-values ≤0.05, **: p-values ≤0.01, p-value: ***≤0.001).

To evaluate the potential role of MIF in inflammation-associated pathogenicity occurring during *T. brucei* infection, two strategies targetting MIF production/activity were evaluated, (i) a comparison between wild type (WT) and MIF-deficient (*Mif* ^−/−^) mice and (ii) a comparison between monoclonal anti-MIF IgG or isotype control antibody treated WT mice. As shown in Fig. 2A, first peak parasitemia and the further progression of parasitemia development were similar in WT and *Mif* ^−/−^ mice. However, there was a small, yet significant prolongation in median survival time in *Mif* ^−/−^ mice (Fig. 2B).

Next, we investigated whether infected WT and *Mif* ^−/−^ mice exhibited different cytokine immune responses. The results shown in Fig. 2C indicate that during the course of infection, both strains of mice mounted a prominent pro-inflammatory immune response as evidenced by the elevated serum levels of IFN-γ, TNF and IL-6. Yet, these cytokine levels were lower in *Mif* ^−/−^ mice than in WT mice, especially during the chronic stage of infection. Conversely, serum IL-10 levels progressively increased during the chronic stage of infection (day 18 p.i. till the end) in *Mif* ^−/−^ mice whereas they were low/marginal in WT mice.

Similar to *Mif* ^−/−^ mice, anti-MIF IgG treatment did not affect parasitemia development but increased the median survival time compared to control antibody treated mice (Fig. S1A-B), suggesting a role for extracellular MIF in disease pathogenesis. As in *Mif* ^−/−^ mice, the pro-inflammatory cytokine production was decreased and IL-10 production was elevated during the chronic stage of infection upon anti-MIF IgG treatment of WT mice (Fig. S1C).

In a tsetse fly-mediated *T. brucei* infection model that mimics the natural route of infection, *Mif* deficiency did not affect parasitemia development but resulted in a prolonged survival (Fig. S2A-B) and a reduced pro-inflammatory cytokine profile (mainly IFN-γ) together with an increased IL-10 production during the chronic stage of infection (Fig. S2C).

### 3. Tissue pathogenicity and infiltration of CD11b^+^Ly6c^high^Ly6G^−^ and CD11b^+^Ly6c^int^Ly6G^+^ myeloid cells are reduced in *Mif^−/−^* mice during the chronic phase of *T. brucei* infection

**Figure 3:**
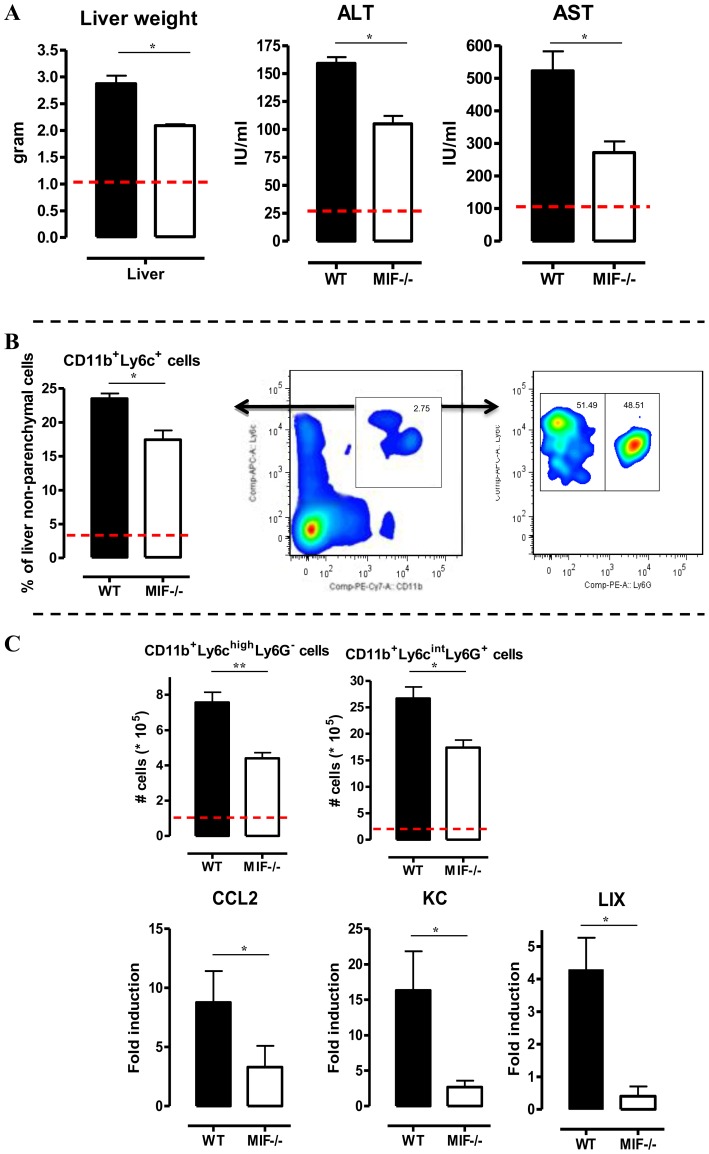
MIF deficiency reduces liver damage and alters liver cell composition during *T. brucei* infection. (A) Liver weight (left panel), serum ALT (middle panel) and AST (right panel) levels in *Mif* ^−/−^ (white bars) and WT (black bars) C57Bl/6 mice at day 25 p.i. (B) Representative liver immune cell gating strategy: Ly6c versus CD11b plot following gating on CD45+ and 7AAD- cells allows identifying CD11b^+^Ly6C^+^ cells (middle panel). This population was plotted in a Ly6C versus Ly6G plot to distinguish CD11b^+^Ly6C^high^Ly6G^−^ inflammatory monocytes and CD11b^+^Ly6C^int^Ly6G^+^ neutrophils (right panel). Percentage of CD11b^+^Ly6C^+^ cells within the liver CD45+ cells in WT (black bars) and *Mif* ^−/−^ (open bars) mice (left panel). (C) Number of CD11b^+^Ly6C^high^Ly6G^−^ and CD11b^+^Ly6C^int^Ly6G^+^ cells within the liver CD45+ cells (upper left panels). The dashed line represents values in non-infected mice. Total liver CCL2, KC (CXCL1) and LIX (CXCL5) gene expression levels in *Mif* ^−/−^ (white bars) and WT (black bars) mice at day 25 p.i. (lower panels). Gene expression levels are normalised using *s12* and expressed relatively to expression levels of non-infected mice. Results are representative of 2 independent experiments and presented as mean of 3 individual mice ± SEM (*: p-values ≤0.05, **: p-values ≤0.01).

A persistent pro-inflammatory immune response contributes to liver damage in the chronic stage of *T. brucei* infection [7], [17]. Interestingly, at this stage (day 25 p.i.), *Mif* ^−/−^ mice exhibited significantly reduced liver pathogenicity than WT mice, as evidenced by lower hepatomegaly and reduced ALT (alanine aminotransferase) levels (Fig. 3A left and middle panel, respectively). The reduced serum AST (aspartate aminotransferase) levels further confirmed lower tissue pathogenicity in infected *Mif* ^−/−^ mice (Fig. 3A right panel).

**Figure 4:**
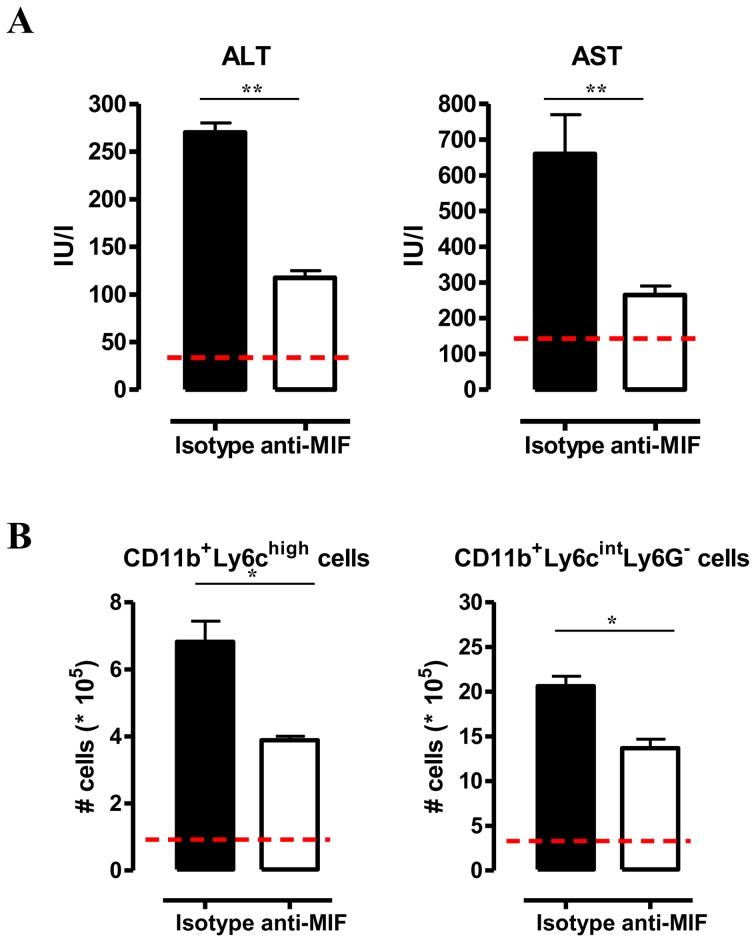
Anti-MIF treatment reduces serum ALT/AST levels and affects liver cell composition during *T. brucei* infection. (A) Serum ALT and AST levels of non-infected (dashed line), isotype control antibody treated (black box) and anti-MIF IgG treated (white box) WT C57Bl/6 mice at day 25 p.i. (B) Total numbers of liver CD11b^+^Ly6C^high^Ly6G^−^ and CD11b^+^Ly6C^int^Ly6G^+^ cells in the chronic (day 25 p.i.) phase of infection calculated using the gating strategy described in Fig. 3B. Results are representative of 2 independent experiments and presented as mean of 3 individual mice ± SEM (*: p-values ≤0.05, **: p-values ≤0.01).

We have documented that infiltration of CD11b^+^Ly6c^+^ myeloid cells in the chronic stage of *T. brucei* infection contributes to liver pathogenicity in WT mice [7], [18]. Upon gating on CD45^+^ liver non-parenchymal cells (see gating strategy Fig. S3A-C), we found that the infiltration of CD11b^+^Ly6c^+^ myeloid cells (Fig. 3B, middle panel) was reduced by 25% in infected *Mif* ^−/−^ mice compared to WT mice (Fig. 3B, left panel). The CD11b^+^Ly6c^+^ cells (Fig. 3B, middle panel) were further subdivided into CD11b^+^Ly6c^high^Ly6G^−^ inflammatory monocytes and CD11b^+^Ly6c^int^Ly6G^+^ neutrophils (Fig. 3B, right panel). Both cell populations were significantly less represented in the liver of infected *Mif* ^−/−^ mice as compared to WT mice (Fig. 3C, upper panels), correlating with a reduced gene expression level of the inflammatory monocyte chemoattractant CCL2 and of the neutrophil chemoattractants CXCL1 (KC) and CXCL5 (LIX) in total liver (Fig. 3C, lower panels). These observations in *Mif* ^−/−^ mice were corroborated by anti-MIF IgG treatment of WT infected mice (Fig. 4A-B).

**Figure 5:**
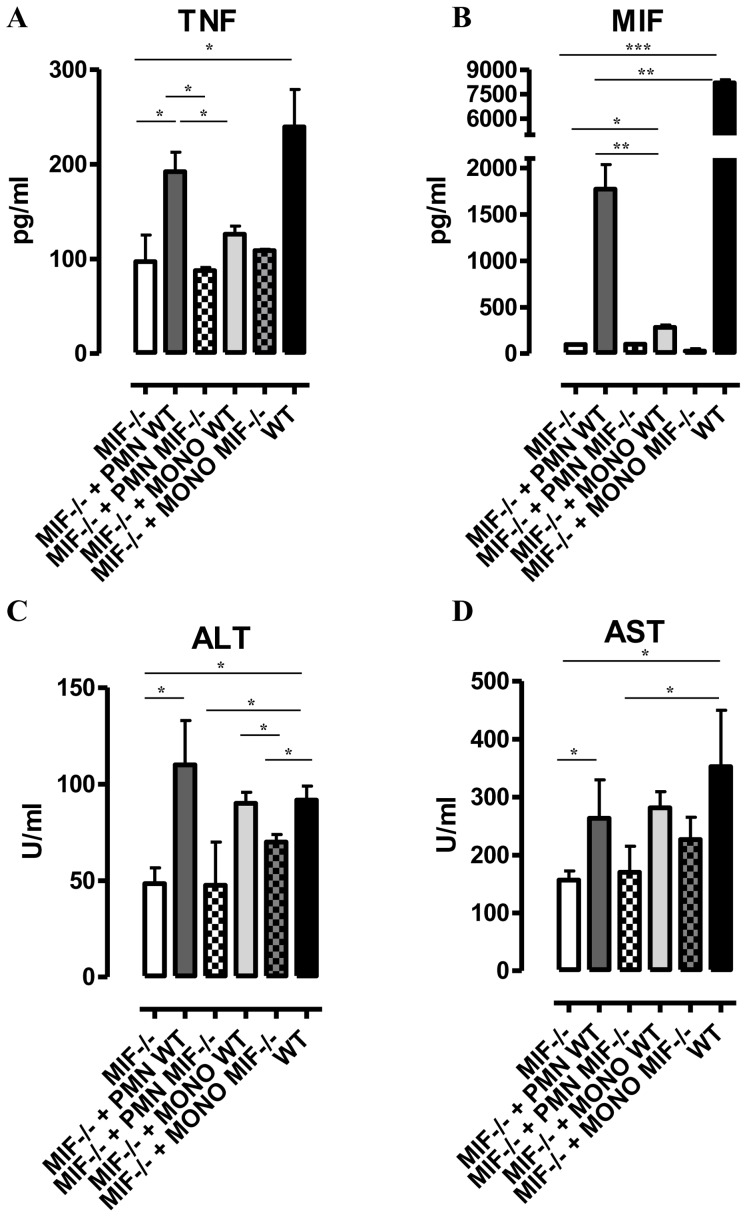
Neutrophil-derived MIF and monocyte-derived MIF contribute to different extent to TNF production and liver injury in *T. brucei* infected mice. TNF and MIF levels from liver cell cultures (A, B), serum ALT (C) and AST (D) levels of infected (day 24 p.i.) *Mif* ^−/−^ mice (white box), of infected *Mif* ^−/−^ mice treated with neutrophils from WT (dark grey box) or *Mif* ^−/−^(white hatched box) infected mice, of infected *Mif* ^−/−^ mice treated with monocytes from WT (light grey box) or *Mif* ^−/−^ (darker grey hatched box) infected mice and of infected WT mice (black box). Results are representative of 3–4 independent experiments and presented as mean of 3 individual mice ± SEM (*: p-values ≤0.05, **: p-values ≤0.01, ***: p-value ≤0.001).

Neutrophils can represent an important source of MIF [19] and so far their contribution to African trypanosomiasis remains unknown. We addressed the possible involvement of neutrophils to *T. brucei* infection outcome in first instance by measuring the myeloperoxidase (MPO) activity as read-out of neutrophil activity. We observed that MPO levels increased more in WT than in *Mif* ^−/−^ mice during the course of infection (Fig. S4). Secondly, we performed adoptive transfer experiments whereby bone marrow-derived neutrophils (i.e. CD11b^+^Ly6c^int^Ly6G^+^ cells) isolated from *T. brucei* infected (day 24 p.i.) WT or *Mif^−/−^* mice were transferred into infected *Mif* ^−/−^ mice. Using neutrophils from *T. brucei* infected ubiquitin-GFP mice we could demonstrate that these cells were still present within the liver of recipient mice 18 hours post-transfer (Fig. S3D-E). Upon transfer of WT but not *Mif* ^−/−^ neutrophils, TNF levels in the liver cell culture supernatants as well as serum ALT/AST levels of *Mif* ^−/−^ recipient mice increased to the levels of infected WT mice (Fig. 5). MIF levels also increased in liver cells culture supernatants of *Mif^−/−^* recipient mice treated with WT neutrophils (Fig. 5), reflecting the contribution of neutrophils to MIF production. On the other hand, adoptive transfer of WT CD11b^+^Ly6c^high^Ly6G^−^ inflammatory monocytes in infected *Mif* ^−/−^ recipient mice resulted in a lower increase in TNF and MIF levels in the liver cell culture supernatants than transfer of WT neutrophils (Fig. S3F-G; Fig. 5). The serum ALT/AST levels increased to a similar level in *Mif^−/−^* recipient mice treated with WT monocytes or WT neutrophils (Fig. 5). Together, these data indicate that during *T. brucei* infection neutrophils can produce more MIF than monocytes and hereby are more important contributors than monocytes to liver pathogenic TNF production. Neutrophils and monocytes contribute to similar extent to liver injury in infected mice. However, while neutrophil-derived MIF is primarily responsible for liver injury, the pathogenic activity of monocytes is relatively less MIF-dependent.

### 4. MIF deficiency correlates with reduced anemia during the chronic phase of *T. brucei* infection

**Figure 6:**
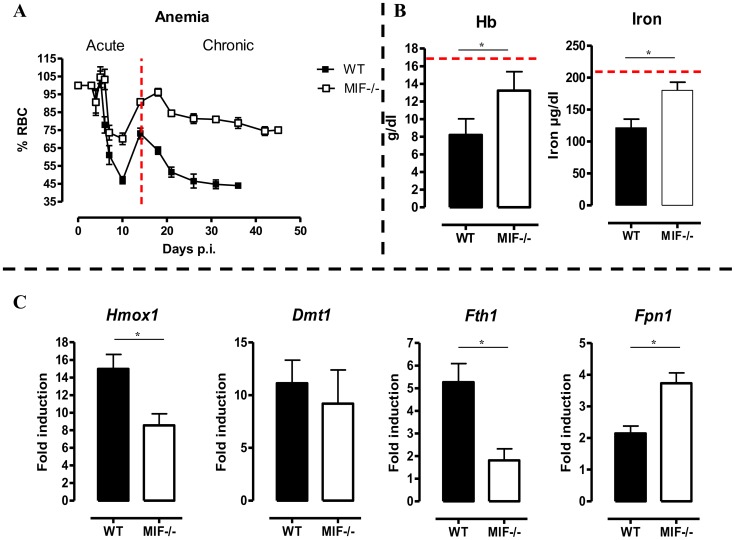
MIF deficiency correlates with reduced anemia, restored hemoglobin/serum iron levels and restored iron homeostasis during *T. brucei* infection. (A) Anemia development during infection in C57Bl/6 (WT, black box; *Mif* ^−/−^, white box) mice. Results are representative of 2–5 independent experiments and expressed as mean of 3–5 individual mice ± SEM. (B) At day 18 p.i., (left panel) hemoglobin levels and (right panel) serum iron levels in *Mif* ^−/−^ (open bars) and WT (black bars) mice. (C) Expression levels of the iron-homeostasis associated genes *Hmox1* (iron import), *Dmt1* (iron transport), *Fth1* (iron storage) and *Fpn1* (iron export) were quantified by RT-QPCR in total livers from *Mif* ^−/−^ (white bars) and WT (black bars) mice at day 18 p.i. Gene-expression levels are normalised using *s12* and expressed relatively to expression levels of non-infected mice. Results are representative of 2 independent experiments and presented as mean of 3 individual mice ± SEM (*: p-values ≤0.05).

Besides liver damage, persistent inflammation during *T. brucei* infection causes anemia [5], which is characterized by two distinct phases; (i) a rapid decline in RBC levels followed by partial recovery during the acute phase of infection and (ii) a more progressive decline in RBC levels during the chronic phase of infection (Fig. 6A). During the acute phase of infection, the initial drop in RBC percentages starts at the same time (day 5–10 p.i.) in both mouse strains but was more severe in WT than *Mif* ^−/−^ mice. Indeed, *Mif* ^−/−^ mice lost about 30% of the total percentage of RBCs while WT mice lost about 50% as compared to non-infected mice (Fig. 6A). Subsequently, a partial RBC recovery phase reaching about 70% of that of non-infected mice occurred (day 10–14 p.i.), whereas in *Mif* ^−/−^ mice this recovery reached about 90% over a longer time period (day 10–18 p.i.). During the chronic phase of infection (following recovery) the RBC levels declined progressively and remained lower in WT than *Mif* ^−/−^ mice. Furthermore, treatment of infected WT mice with anti-MIF IgG resulted in a better recovery of RBCs similar as in infected *Mif* ^−/−^ mice (Fig. S5A). Finally, *Mif* ^−/−^ mice also showed reduced anemia upon tsetse fly-based infection (Fig. S5B).

### 5. *Mif* ^−/−^ mice exhibit restored iron homeostasis during the chronic phase of *T. brucei* infection

Anemia development during persistent inflammation can result from iron accumulation within the mononuclear phagocyte system (MPS) leading to iron deprivation from erythropoiesis. Hence, the reduced pro-inflammatory immune response in *Mif* ^−/−^ mice during the chronic stage of *T. brucei* infection as compared to WT mice might impact on iron-homeostasis and hemoglobin levels. At day 18 p.i., the time-point when the differences in anemia development and cytokine levels between WT and *Mif* ^−/−^ mice become apparent (see Fig. 6A and Fig. 2C), hemoglobin and serum iron levels were less reduced in *Mif* ^−/−^ mice as compared to WT mice (Fig. 6B). These differences were corroborated at the level of expression of genes implicated in iron homeostasis in the liver. In this context, it should be emphasized that under physiological conditions, tissue-associated myeloid cells, in particular liver myeloid cells, recover ferrous iron (Fe^2+^) via engulfment of senescent RBCs and hemoglobin recycling. The Fe^2+^ iron from hemoglobin is extracted by myeloid cells via heme oxygenase-1 (HO-1), then transported into the cytosol by the divalent metal transporter 1 (DMT-1/Nramp2), from where it can be either exported from or stored inside the myeloid cells via ferroportin-1 (FPN-1) or ferritin (FHC), respectively, to prevent toxicity, depending on the iron demand of the host [6]. As shown in Fig. 6C, there was a lower liver HO-1 (*Hmox1*) gene expression level in infected *Mif* ^−/−^ than WT mice (suggesting lower iron extraction), while the DMT-1 (*Dmt1*) gene expression levels were similar in both mouse groups (suggesting similar iron transport). In addition, the *FHC* (*Fth1*) gene expression levels were lower in infected *Mif* ^−/−^ mice (Fig. 6C), indicating reduced iron accumulation in *Mif* ^−/−^ than WT mice. Finally, *Fpn1* mRNA levels were higher in infected *Mif* ^−/−^ mice (Fig. 6C), suggesting that iron export in these mice is less impaired as compared to WT mice. Together these data indicate that there is less iron extracted and retained in the MPS from infected *Mif* ^−/−^ than WT mice, which in turn allows increased iron availability for erythropoiesis.

### 6. *Mif^−/−^* mice display enhanced erythropoiesis during the chronic phase of *T. brucei* infection

**Figure 7:**
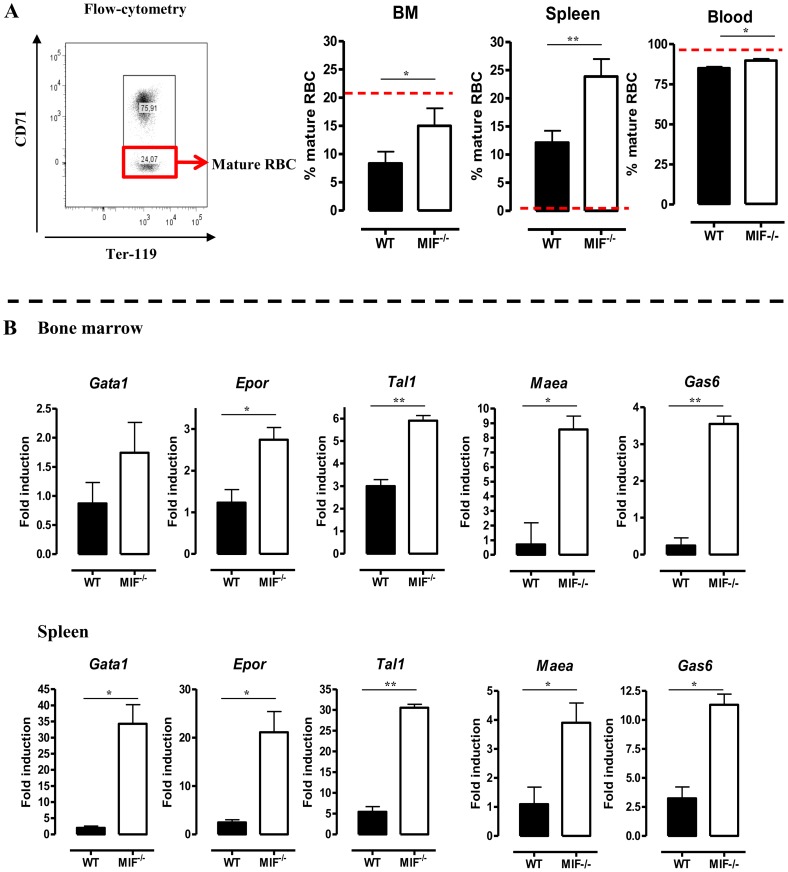
MIF deficiency correlates with restored/enhanced erythropoiesis during *T. brucei* infection. (A) Representative profile for the gating of mature (Ter-119^+^ CD71^−^) and immature (Ter-119^+^ CD71^+^) RBCs in bone marrow of non-infected C57Bl/6 mice (left panel). The percentage of mature RBCs within the total Ter-119^+^ population in bone marrow, spleen and blood at day 18 p.i. (right panels). Dashed line represents percentage of mature RBCs in non-infected mice, which was similar in WT and *Mif−/−* mice. Results are representative of 3 independent experiments and shown as mean of 3 individual mice ± SEM. (B) Expression levels of genes involved in erythropoiesis in total bone marrow (upper panels) and spleen (lower panels) from *Mif* ^−/−^ (white bars) and WT (black bars) mice at day 18 p.i. Gene-expression levels are normalised using *s12* and expressed relatively to expression levels of non-infected mice. Results are representative of 2 independent experiments and presented as mean of 3 individual mice ± SEM (*: p-values ≤0.05, **: p-values ≤0.01).

**Figure 8:**
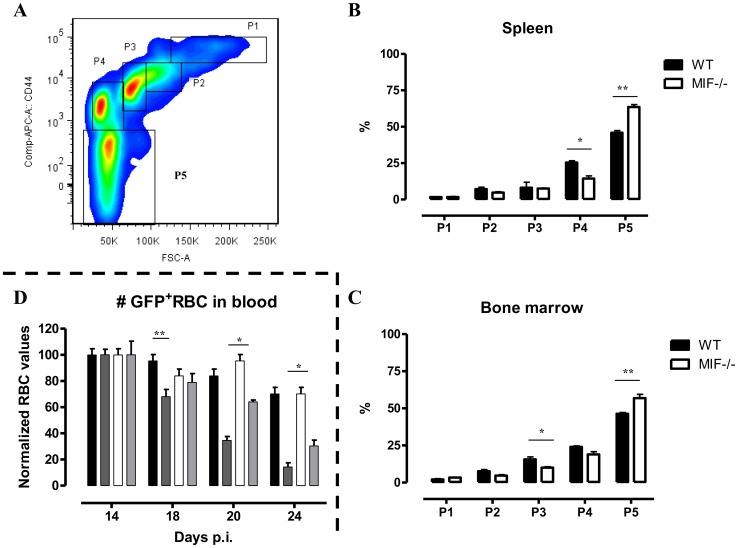
MIF deficiency correlates with enhanced terminal RBC differentiation and reduced RBC clearance during *T. brucei* infection. (A) Gating strategy used to discriminate different stages of erythroid development, starting from nucleated erythroblasts (P1 (pro), P2 (Basophilic + polychromatic) and P3 (orthochromatic)) till enucleated erythrocytes (P4 (reticulocyte) and P5 (erythrocyte)). 7AAD^−^CD45^−^Ter-119+ cells were selected and plotted in a CD44 versus FSC plot (upper panel). The percentage of the different erythroid populations at day 18 p.i. in WT (black box) and *Mif* ^−/−^ (white box) mice is shown for the spleen (B) and bone marrow (C). Results are representative of 2 independent experiments and presented as mean of 3 individual mice ± SEM. (D) RBC clearance in non-infected (WT, black bars; *Mif* ^−/−^, white bars) and day 12 p.i. *T. brucei* infected (WT, dark grey bars; *Mif* ^−/−^, light grey bars) C57Bl/6 mice following i.v injection with 200 µl GFP^+^ RBCs. At different time points after injection the presence of GFP^+^ RBCs following gating on Ter-119^+^ cells in the blood was evaluated. GFP^+^ RBC numbers were normalized, whereby GFP^+^ RBCs present after 1 day post infection are being referred as 100%. Results are representative of 2 independent experiments and presented as mean of 5 individual mice ± SEM (*: p-values ≤0.05, **: p-values ≤0.01).

The persistent pro-inflammatory immune response in *T. brucei* infection (see Fig. 2C), leading to iron accumulation within the MPS and iron deprivation (see Fig. 6B-C) could impair erythropoiesis. Therefore, the efficiency of the constitutive (*i.e.* bone marrow) and inflammation-induced (i.e. spleen) erythropoiesis was determined in WT and *Mif* ^−/−^ mice during the chronic stage of infection (day 18 p.i.) using three different approaches. First, as shown in Fig. 7A (left panel) we measured the relative abundance of immature (Ter-119^+^CD71^+^) and mature (Ter-119^+^CD71^−^) RBCs as described by [20]. Infected *Mif* ^−/−^ mice had relatively more mature RBCs in the bone marrow and the spleen than WT mice (Fig. 7A, right panels). Concomitantly, the reduction in the percentage of mature RBCs in the blood was less pronounced in infected *Mif* ^−/−^ mice than in WT mice. Secondly, we determined the expression levels of representative genes involved in RBC differentiation (*Gata1*, *Epor*, *Tal1*, *Maea* and *Gas6*) in the bone marrow and spleen. As shown in Fig. 7B, the expression of most genes was not induced (with the exception of *Tal1*) during infection in WT mice in both organs, while they increased in infected *Mif* ^−/−^ mice. Thirdly, we quantified the different stages of erythropoiesis (from nucleated erythroblasts (P1) till enucleated erythrocytes (P5)) using a recently described protocol [21] based on a CD44 versus FSC profile following gating on the Ter-119^+^ cells (Fig. 8A). It appeared that compared to WT mice, infected *Mif* ^−/−^ mice had a more efficient RBC maturation mainly at the later stage of differentiation (reflected by the increased percentage of P5) in the spleen and to a lesser extent in the bone marrow (Fig. 8B-C, respectively). Together, these data demonstrate a higher level of erythropoiesis in the absence of MIF in *T. brucei* infected mice.

### 7. *Mif* ^−/−^ mice display a reduced RBC clearance during the chronic phase of *T. brucei* infection

An increased RBC elimination may also contribute to anemia in *T. brucei* infected mice. To address this question, we injected green fluorescent protein positive (GFP^+^) RBCs i.v. at the start of the chronic phase of *T. brucei* infection (day 12 p.i.) in WT and *Mif* ^−/−^ mice and analyzed their clearance as the infection progressed. Non-infected WT and *Mif* ^−/−^ mice were treated similarly with GFP^+^ RBCs as controls. As shown in Fig. 8D, there was no difference in GFP^+^ RBC clearance between non-infected WT and *Mif* ^−/−^ mice. However, the GFP^+^ RBC clearance during infection was faster compared to non-infected mice. In addition, relatively more GFP^+^ RBCs remained in infected *Mif* ^−/−^ compared to WT mice (Fig. 8D). These data suggest that a reduction in RBC elimination observed in *Mif* ^−/−^ mice could contribute to less severe anemia during *T. brucei* infection.

## Discussion

We investigated herein the role of MIF, an upstream regulator of the inflammatory response, in the immunopathogenicity of experimental African trypanosomiasis. We found that during *T. brucei* infection in trypanosusceptible wild type (WT) mice, the MIF gene expression profile in lymphoid tissues and the systemic serum protein level consists of two distinct waves; an upregulation during the acute phase of infection that declines after the first peak of parasitemia, followed by a second increase as the infection progresses to the chronic phase. Our results are in line with observations documenting that the *Mif* mRNA levels were increased in spleen cells during *T. brucei* infection in rats [22]. Using *Mif* ^−/−^ mice and neutralizing anti-MIF IgG treatment, we revealed that absence/blockade of MIF did not affect parasite growth. Yet, absence of MIF slightly increased survival time and reduced serum IFN-γ, TNF, and IL-6 concentrations while inducing IL-10 production in the chronic stage of infection. This observation most likely reflects a switch from a pro-inflammatory/pathogenic type I to an anti-inflammatory/anti-pathogenic type 2 response in the host. The higher TNF and IFN-γ response observed in WT mice compared to *Mif* ^−/−^ mice during the chronic stage of infection could result from the reported positive feedback loop between MIF, TNF and IFN-γ [23].

The switch from a pro-inflammatory to an anti-inflammatory immune response in *Mif* ^−/−^ or anti-MIF IgG treated WT mice during the chronic stage of infection was reflected by a reduction in pathogenicity, as evidenced by less liver damage and anemia, which are the main pathogenic manifestations of *T. brucei* infection [3], [7]. CD11b^+^Ly6c^high^Ly6G^−^ inflammatory monocytes are reported to contribute to *T. brucei*-induced pathogenicity development through their production of TNF, whereby in the acute phase of infection their emigration from the bone marrow is CCL2/CCR2-dependent [18], [24]. Although MIF can induce CCL2 [25] and trigger monocyte recruitment and subsequent arrest in tissue [14], the bone marrow emigration and recruitment of inflammatory monocytes in tissues during the acute stage of infection was found independent of MIF [24]. We now report that MIF contributed to the recruitment of inflammatory monocytes into the liver in the chronic stage of *T. brucei* infection. The lower accumulation of inflammatory monocytes in *Mif* ^−/−^ mice at this stage of infection could result from the reduced expression of *Ccl2* and/or increased production of IL-10. Indeed, during *T. brucei* infection, IL-10 has been reported to limit the CCR2-mediated egress of monocytes from the bone marrow and to suppress their differentiation/maturation into TNF producing cells, thereby preventing tissue damage [18], [24]. The more sustained MIF production during the chronic stage of infection compared to the acute stage could also account for the observed accumulation of inflammatory monocytes in WT infected mice.

Our data further revealed that CD11b^+^Ly6c^int^Ly6G^+^ neutrophils accumulated in the liver of WT mice during the chronic stage of *T. brucei* infection, coinciding with increased gene expression of the neutrophil-specific chemokines KC and LIX in total liver [26]. Yet, this increase was higher in WT than *Mif* ^−/−^ mice. Of note, besides indirect effects of MIF on monocyte and neutrophil recruitment mediated via induction of CCL2 and KC/LIX, respectively, we can't exclude that direct MIF effects account for the observed difference in cell recruitment/retention between WT and *Mif* ^−/−^ infected mice. Indeed, MIF features structural motives shared by canonical CXC+ ligands and is therefore considered a non-cognate ligand of the chemokine receptors CXCR2 and CXCR4 that, through interaction with these receptors, can be instrumental in inflammatory pathogenic leukocyte recruitment [14]. Moreover, MIF was also shown to bind CD74 (Ia-associated invariant chain), a single-pass membrane-receptor, which in turn can form functional complexes with either CXCR2 or CXCR4 and subsequently mediates MIF-specific signaling [14].

Although neutrophilia has been documented before [27], the role of neutrophils in the outcome and pathogenesis of African trypanosomiasis is poorly investigated. In this regard, we report that the serum myeloperoxidase activity, as indicator of neutrophil activity which can play a pro-inflammatory pathogenic role during chronic infections [28], increased significantly during *T. brucei* infection in WT mice and to a lower extent in *Mif* ^−/−^ mice. An accumulation of neutrophils in the liver of *T. brucei* infected mice can be relevant. Indeed, neutrophils represent an important source of MIF that can be released upon activation or TNF-induced apoptosis [19]. Moreover, neutrophils can enhance hepatocyte death that release necrotic products into the circulation triggering a systemic inflammatory immune response [29], which is the major cause of tissue pathogenicity in *T. brucei* infected mice. Accordingly, liver MIF protein levels increased drastically during the chronic phase of infection. Moreover, we observed a pathogenic role for WT neutrophils upon their adoptive transfer into infected *Mif* ^−/−^ recipient mice, which resulted in increased TNF production and increased ALT/AST levels. Since the transfer of neutrophils from *Mif* ^−/−^ mice into WT recipient mice did not affect TNF and liver injury, it seems that MIF production by neutrophils evidenced in this report plays a prominent, previously unappreciated role in trypanosomiasis-associated pathogenicity. On the other hand, the transfer of WT monocytes, that contribute to MIF production in acute *T. brucei* infection stage [24], into *Mif* ^−/−^ mice recipient mice only resulted in a moderate increase in MIF and TNF production but induced similar level of tissue injury than the transfer of WT neutrophils. Hence, neutrophil-derived MIF is a more important contributor than monocyte-derived MIF to pathogenicity in infected mice. To our knowledge, this is the first time that the neutrophil is identified as the more prominent source of MIF in a disease situation. Although the pathogenic activity of monocytes is relatively less dependent from their MIF production and TNF induction than the one of neutrophils, monocytes could contribute to liver injury through production of other pathogenic type I immune response associated molecules such as Cxcl10, Cxcl9 or Ccl3 [24].

Besides liver pathogenicity, a clinically significant anemia develops during African trypanosomiasis [3], [30]. We observed reduced anemia and preserved serum iron levels during the course of *T. brucei* infection in *Mif* ^−/−^ mice. These data are in concordance with our previous results showing that reducing the inflammatory immune responses using a GPI-based treatment coincides with the alleviation of anemia and improved iron-homeostasis resulting in higher iron-bioavailability and increased erythropoiesis [31]. Similarly, key genes involved in iron homeostasis were differentially modulated in *T. brucei* infected WT and *Mif* ^−/−^ mice. Indeed, *Hmox1* which is involved in RBC catabolism and iron extraction from hemoglobin, together with the iron storage molecule *Fth1*, showed reduced expression in infected *Mif* ^−/−^ mice. Conversely, the iron cell exporter *Fpn1* expression increased upon *T. brucei* infection. Given that TNF and IFN-γ can induce *Hmox1*, that TNF and IL-6 are important inducers of *Fth1* and that IFN-γ can suppress *Fpn1* expression [6], the reduced levels of TNF and IL-6, as well as of IFN-γ may explain for the lower *Hmox1*, *Fth1* and higher *Fpn1* expression levels in *Mif* ^−/−^ mice.

The reduced inflammatory immune response coupled with restored iron-homeostasis in infected *Mif* ^−/−^ mice could alleviate the suppression of erythropoiesis that occurs during the chronic phase of *T. brucei* infection [32]. Our data indeed show that the absence of MIF correlated with improved erythropoiesis as evidenced by the higher percentage of mature RBCs and increased expression of genes involved in erythropoiesis (*Gata1*, *Epor*, *Tal1*, *Maea* and *Gas6*) in both the bone marrow and spleen during the chronic phase of infection. In this context, elevated MIF levels in the spleen and bone marrow of *P. chabaudi*-infected mice were found to inhibit the early stages of erythropoiesis as well as hemoglobin production [33]. In the *T. brucei* African trypanosomiasis model, the suppressive effect of MIF was observed at the later stage of erythroid differentiation. The increased gene expression levels in *T. brucei* infected *Mif* ^−/−^ mice of *Maea*, which is crucial for the enucleation of reticulocytes [34], and of *Gas6*, which reduces TNF-mediated apoptosis of erythroid progenitor cells [35], may contribute to the more efficient RBC maturation. The reduced anemia observed in *T. brucei* infected *Mif* ^−/−^ mice could also be attributed to the better RBC recovery/reduced RBC clearance in the chronic phase of infection, which in turn could result from reduced myeloid cell activation and/or lower IFN-γ as compared to WT infected mice.

Collectively, our results suggest that in the chronic phase of *T. brucei* infection, MIF contributed to inflammation-associated pathogenicity by (i) sustaining a persistent pro-inflammatory type I immune response and (ii) maintaining/enhancing the recruitment of pathogenic monocytic cells and neutrophils in the liver. Hereby, neutrophil-derived MIF contributed significantly to enhanced TNF production and liver damage. In addition, MIF contributed to (iii) iron-accumulation in liver myeloid cells, suppressing erythropoiesis at later stages of erythroblast differentiation and (iv) enhanced RBC clearance. Despite reduced pathogenicity, *Mif* ^−/−^ mice exhibit only a moderate increase in survival compared to WT mice, inferring that MIF-independent mechanisms determine survival of infected mice. Yet, so far the exact cause of death in murine African trypanosomiasis is unknown and is suggested to be due to Systemic Inflammatory Response Syndrome (SIRS), thus likely multifactorial [36] and going beyond the role of MIF.

Interestingly, polymorphisms in the *Mif* gene have been shown to contribute to susceptibility in several inflammatory diseases (reviewed in [37]). Moreover, low-expression *MIF* alleles, which occur more commonly in Africans, may offer protection from disease manifestations in trypanosomiasis endemic settings [38]. Therefore, interfering with MIF signaling could be a novel approach to limit inflammation-associated complications during *T. brucei* trypanosomiasis.

## Materials and Methods

### Ethics statement

All experiments complied with the ECPVA guidelines (CETS n° 123) and were approved by the VUB Ethical Committee (Permit Number: 08-220-8). *T. brucei* tsetse fly infections were approved by the Environmental administration of the Flemish government.

### Parasites, mice and infections

Clonal pleomorphic *T. brucei* AnTat 1.1E parasites were a kind gift from N. Van Meirvenne (Institute for Tropical Medicine, Belgium) and stored at −80°C. Tsetse flies infected with non-clonal *T. brucei* AnTAR1 parasites were maintained at the Institute of Tropical Medicine. Wild type (WT) C57Bl/6 mice were obtained from Janvier. MIF deficient (*Mif* ^−/−^) [39] and ubiquitin-GFP (Jackson Laboratories) C57Bl/6 mice were bred in our animal facility.

Female mice (7–8 weeks old) were infected with 5×10^3^ AnTat1.1E trypanosomes (intraperitonealy (i.p.)) or using one individual tsetse fly with a mature salivary gland infection which was allowed to feed per mouse.

Parasite and red blood cell (RBC) numbers in blood were determined via haemocytometer by tail-cut (2.5 µl blood in 500 µl RPMI). Anemia was expressed as percentage of RBCs remaining in infected mice compared to that of non-infected mice. MIF-neutralisation experiments were performed by i.p. injection of mice with 500 µg neutralizing anti-MIF IgG1 [40] or matching isotype control IgG (University of Louvain, Experimental Immunology Unit) every second day starting from day 1 post infection (p.i.).

### Measurement of serum iron and myeloperoxidase (MPO) content as well as of blood hemoglobin levels

Total serum iron was measured using the IRON FZ kit (Chema Diagnostics) as recommended by the suppliers. Serum MPO concentrations were measured as described by the suppliers (R&D systems). Blood Hemoglobin levels were quantified colorimetrically. Briefly, 2 µl blood was diluted in 200 µl distilled water in a 96 well round bottom plate (Falcon) and incubated for 30 min. at 37°C. After centrifugation (600 g, 10 min.) the supernatant was collected and the OD540nm measured in an Ultra Microplate reader (ELx808, Bio-Tek instruments.inc). The hemoglobin concentration was calculated using a hemoglobin standard (Sigma).

### Liver cell isolation protocol

Livers from CO_2_ euthanized mice were perfused with 30 ml heparinized saline (10 units/ml; Leo Pharma) containing 0.05% collagenase type II (*Clostridium histolyticum*; Sigma-Aldrich), excised and rinsed in saline. Following liver mincing in 10 ml digestive media (0.05% collagenase type II in Hanks' Balanced Salt Solution (HBSS) without calcium or magnesium; Invitrogen) and incubation at 37°C for 30 min, the digested tissue was homogenized and filtered (40 µm pore filter). The cell suspension was centrifuged (7 min, 300×*g*, 4°C) and the pellet treated with erythrocyte-lysis buffer. Following centrifugation (7 min, 300×*g*, 4°C) the pellet was resuspended in 2–5 ml RPMI/5%FCS and cells counted to bring them at 10^7^ cells/ml for flow-cytometric analysis, RT-QPCR and cell culturing. For RT-QPCR analysis 10^7^ cells were resuspended in 1 ml TRIzol (Gibco-Invitrogen Life Technologies) and stored at −80°C.

### Spleen and bone marrow cell isolation

Spleen and bone marrow (tibia and femur) cells were obtained by homogenizing the organs in 10 ml RPMI medium, passing the suspension through a 40 µm pore filter and centrifugation (7 min, 300×*g*, 4°C). After resuspending the pellet, cells were counted and brought at 10^7^ cells/ml for flow cytometry (RBC) analysis. Remaining cells were pelleted (7 min, 300×*g*, 4°C) and processed as described for the liver (see above).

### Cytokine analysis

Concentrations of MIF and TNF (R&D Systems) as well as IFN-γ, IL-6 and IL-10 (Pharmingen) in serum and/or culture medium were determined by ELISA as recommended by the suppliers.

### Real-time quantitative polymerase chain reaction (RT-QPCR) analysis

1 µg total RNA prepared from 10^7^ cells was reverse-transcribed using oligo(dT) and Superscript II Reverse Transcription (Roche Molecular Systems) following the manufacturer's recommendations. RT-QPCR conditions were as described in [41]. Primer sequences are reported in Table S1 and Ct values of the *S12* household gene for naive and infected mice samples (within the BM, spleen and liver) is shown in Table S2.

### Flow cytometry

Blood, spleen and bone marrow cells were analysed before RBC lysis. Briefly, total blood (2.5 µl diluted in 500 µl RPMI) and spleen and bone marrow (100 µl from a stock solution of 10^7^ cells/ml) were incubated (20 min, 4°C) with Fc-gamma blocking antibody (2.4G2, BD Biosciences) and further stained with phycoerythrin (PE)-conjugated anti-Ter-119, fluorescent isothiocyanate (FITC)-conjugated anti-CD71, Allophycocyanin (APC)-conjugated CD44 (eBioscience, ImmunoSource), PE-Cy7 conjugated rat anti-CD11b (BD Pharmingen) and matching control antibodies. The cells were washed with PBS, measured on FACSCanto II (BD Biosciences) and the results analysed using FlowJo software by gating on Ter-119^+^ cells.

The bone marrow, spleen and liver cells after RBC lysis were analyzed using APC-Cy7 conjugated CD45 (BD Pharmingen), PE-Cy7 conjugated rat anti-CD11b (BD Pharmingen), Alexa647-conjugated rat anti-Ly6c (Serotec), PE-conjugated rat anti-Ly6G (BD Pharmingen) and matching control antibodies. 7AAD (BD Pharmingen) was used to exclude death cells.

### Adoptive transfer experiments

Neutrophils and monocytes were isolated from the bone marrow of infected mice (day 24 p.i). Following isolation and surface staining as described above using PE-Cy7 conjugated rat anti-CD11b (BD Pharmingen) and Alexa647-conjugated rat anti-Ly6c (Serotec), neutrophils were sorted based on their CD11b^+^Ly6c^int^ surface expression and FSC/SSC profile using FACSAria II (BD) and subsequently stained with PE-conjugated rat anti-Ly6G (BD Pharmingen) to confirm their purity (95–99%). Monocytes were sorted based on their CD11b^+^Ly6c^high^ surface expression and FSC/SSC profile using FACSAria II (BD). Next, 3 individual infected (24 days p.i.) WT or *Mif* ^−/−^ mice were injected intravenously (i.v.) with 2×10^6^ neutrophils, 2×10^6^ monocytes or 200 µl RPMI (control group). 18 hours after transfer cells were isolated from the liver of recipient mice and analyzed.

### Cell culturing

Liver cells (stock: 10^7^/ml) were diluted till 2×10^6^ cells/ml in RPMI-1640 medium/10% FCS/non-essential amino acids/glutamate/penicillin/streptomycin. Next, 500 µl cell suspension/well were cultured (36 hours, 37°C, 5% CO_2_) in 48 well plates (Nunc) and the supernatant tested for TNF levels.

### Aspartate transaminase (AST) and alanine transaminase (ALT) measurement

Serum AST and ALT levels were determined as described by the suppliers (Boehringer Mannheim Diagnostics).

### RBC clearance assay

200 µl heparinized blood from 7–8 weeks old ubiquitin-GFP C57Bl/6 mice was injected i.v. into 3–5 non-infected or infected (12 days p.i.) mice. Every second day, RBC numbers were enumerated via haemocytometer (see above) and the remaining cells analysed via FACS (see above) using PE-conjugated Ter-119 antibody. Following gating on Ter-119^+^ cells, two distinct RBC populations could be identified (GFP+ or -). To calculate the RBC clearance, the percentage of GFP^+^ RBCs present at day 1 post injection was referred to as 100% signal. All other time-points were compared with this day.

### Statistical analysis

The GraphPad Prism 4.0 software was used for statistical analyses (Two-way ANOVA or student *t*-test). Values are expressed as mean ± SEM. Values of p≤0.05 are considered statistically significant.

## Supporting Information

Figure S1 Anti-MIF treatment confers protection and reduces inflammatory immune responses during *T. brucei* infection. (A) Parasitemia and (B) survival in C57Bl/6 mice following isotype control (black triangle) or anti-MIF IgG (inverted white triangle) treatment. Results are representative of 3 independent experiments and presented as mean of 3 individual mice ± SEM. (C) Serum cytokine levels of IFN-γ (upper left panel), TNF (upper right panel), IL-6 (lower left panel) and IL-10 (lower right panel) in isotype treated (dark grey) and anti-MIF IgG treated (light gray) WT mice at day 11 and 25 p.i. Results are representative of 2 independent experiments and presented as mean of 3 individual mice ± SEM (*: p-values ≤0.05).
(TIF)

Figure S2 MIF deficiency confers protection and reduces inflammatory immune responses during tsetse fly (*AnTar1*) mediated infection. (A) Parasitemia and (B) survival in C57Bl/6 (WT, black box) and MIF deficient (*Mif* ^−/−^, white box) mice. (C) Serum cytokine levels of IFN-γ (upper left panel), TNF (upper right panel), IL-6 (lower left panel) and IL-10 (lower right panel) in *Mif* ^−/−^ (white bars) and WT (black bars) C57Bl/6 mice. Results are representative of 2–3 independent experiments and presented as mean of 3–5 individual mice ± SEM (*: p-values ≤0.05, **: p-values ≤0.01).
(TIF)

Figure S3 Representative liver gating strategy for neutrophils and monocytes. Selection of (A) live gate based on the FSC-A/SSC-A profile of total liver cells from *T. brucei* infected WT mice (day 24 p.i.); (B) of single cells within the life gate based on the SSC-A/FSC-W profile; (C) of CD45^+^ cells within the single cell gate based on a FSC-A/CD45 profile. (D) CD11b versus GFP profile within the CD45^+^ population allows detection of GFP^+^ cells following adoptive transfer of CD11b^+^Ly6c^int^Ly6G^+^ cells from ubiquitin-GFP mice. (E) CD11b versus GFP profile within the CD45^+^ population from control mice receiving medium. (F) CD11b versus GFP profile within the CD45^+^ population allows detection of GFP^+^ cells following adoptive transfer of CD11b^+^Ly6c^high^Ly6G^−^ cells from ubiquitin-GFP mice. (G) CD11b versus GFP profile within the CD45^+^ population from control mice receiving medium.
(TIF)

Figure S4 Serum myeloperoxidase activity increases during *T. brucei* infection. Fold increase in serum myeloperoxidase (MPO) in WT (black box) and *Mif* ^−/−^ (white box) C57Bl/6 mice (compared to levels in non-infected mice, dashed line). Results are representative of at least 2 independent experiments and expressed as mean of 5 individual mice ± SEM. (*: p-values ≤0.05, **: p-values ≤0.01, ***: p-values ≤0.001).
(TIF)

Figure S5 MIF deficiency reduces anemia during anti-MIF treatment and tsetse fly mediated *T. brucei* infection. (A) Anemia levels following i.p. injection of isotype control (black triangle) and anti-MIF IgG treated (inverted white triangle) WT mice. (B) Anemia development during tsetse fly-based infection (AnTar1) infection in C57Bl/6 (WT) (black box) and *Mif* ^−/−^ (white box) mice. Results are representative of at least 3 independent experiments and expressed as mean of 5 individual mice ± SEM.
(TIF)

Table S1 Primers used for RT-QPCR analysis. Table summarizing the gene names and corresponding primer sequences of all primers used in this manuscript. All primers were purchased at Sigma and designed using the Primer-Blast software (NCBI).
(DOCX)

Table S2 Ct values via RT-PRC for the household gene *s12* of naïve and infected mice. Overview of the Ct values for the household gene *s12* for naïve and *T. brucei* infected mice at the level of the bone marrow, spleen and liver. Note: Results are representative of 2–3 independent experiments and presented as mean of 3 individual mice ± SD.
(DOCX)
