# Supplementary figures and images for: MIF Contributes to Trypanosoma brucei Associated Immunopathogenicity Development
Source: PLoS Pathog. 2014 Sep 25;10(9):e1004414. doi: 10.1371/journal.ppat.1004414 (PMC4177988; doi:10.1371/journal.ppat.1004414)

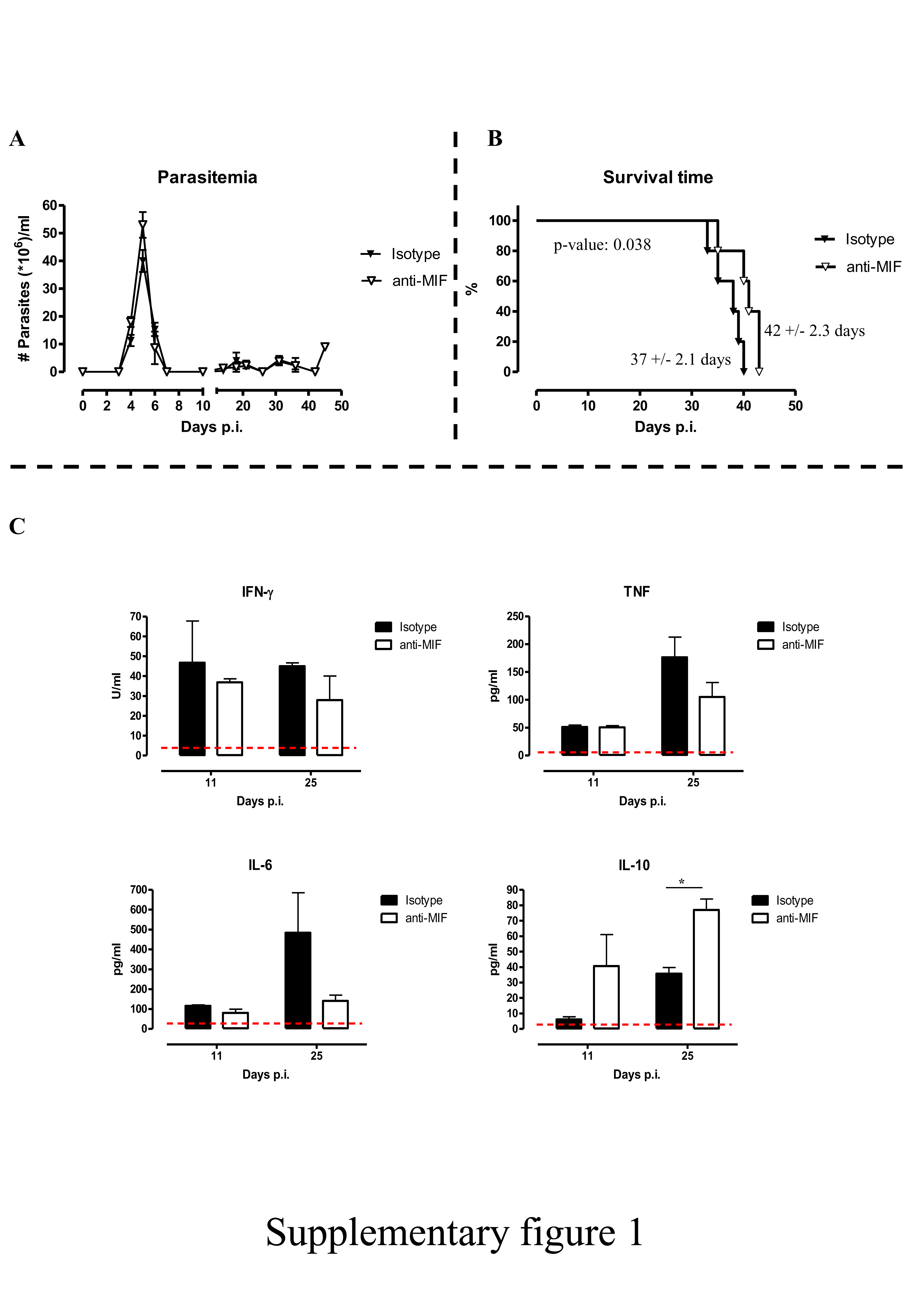

Supplement: Figure S1 — Anti-MIF treatment confers protection and reduces inflammatory immune responses during T. brucei infection. (A) Parasitemia and (B) survival in C57Bl/6 mice following isotype control (black triangle) or anti-MIF IgG (inverted white triangle) treatment. Results are representative of 3 independent experiments and presented as mean of 3 individual mice ± SEM. (C) Serum cytokine levels of IFN-γ (upper left panel), TNF (upper right panel), IL-6 (lower left panel) and IL-10 (lower right panel) in isotype treated (dark grey) and anti-MIF IgG treated (light gray) WT mice at day 11 and 25 p.i. Results are representative of 2 independent experiments and presented as mean of 3 individual mice ± SEM (*: p-values ≤0.05). (TIF) [file ppat.1004414.s001.tif]

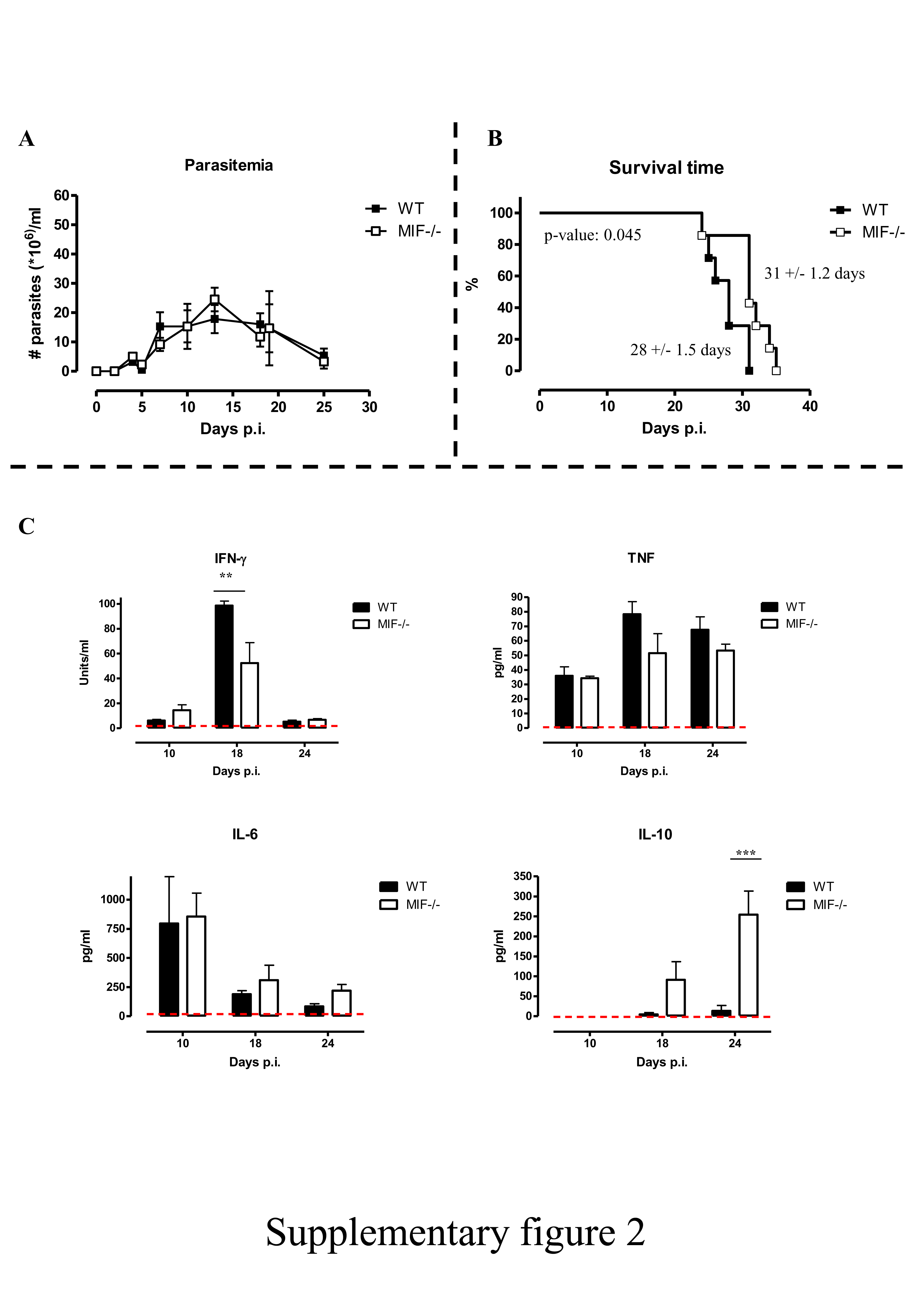

Supplement: Figure S2 — MIF deficiency confers protection and reduces inflammatory immune responses during tsetse fly (AnTar1) mediated infection. (A) Parasitemia and (B) survival in C57Bl/6 (WT, black box) and MIF deficient (Mif −/−, white box) mice. (C) Serum cytokine levels of IFN-γ (upper left panel), TNF (upper right panel), IL-6 (lower left panel) and IL-10 (lower right panel) in Mif −/− (white bars) and WT (black bars) C57Bl/6 mice. Results are representative of 2–3 independent experiments and presented as mean of 3–5 individual mice ± SEM (*: p-values ≤0.05, **: p-values ≤0.01). (TIF) [file ppat.1004414.s002.tif]

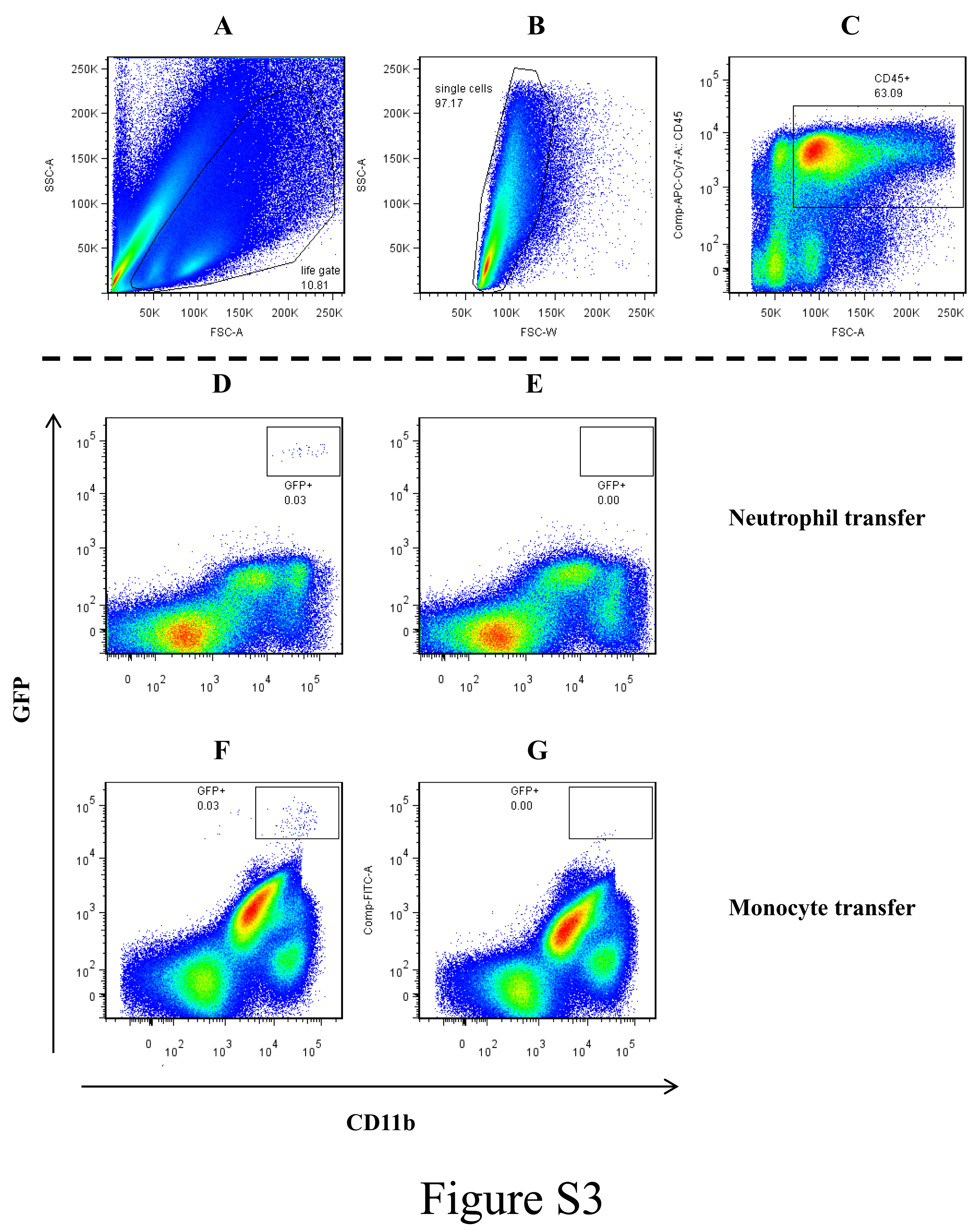

Supplement: Figure S3 — Representative liver gating strategy for neutrophils and monocytes. Selection of (A) live gate based on the FSC-A/SSC-A profile of total liver cells from T. brucei infected WT mice (day 24 p.i.); (B) of single cells within the life gate based on the SSC-A/FSC-W profile; (C) of CD45+ cells within the single cell gate based on a FSC-A/CD45 profile. (D) CD11b versus GFP profile within the CD45+ population allows detection of GFP+ cells following adoptive transfer of CD11b+Ly6cintLy6G+ cells from ubiquitin-GFP mice. (E) CD11b versus GFP profile within the CD45+ population from control mice receiving medium. (F) CD11b versus GFP profile within the CD45+ population allows detection of GFP+ cells following adoptive transfer of CD11b+Ly6chighLy6G− cells from ubiquitin-GFP mice. (G) CD11b versus GFP profile within the CD45+ population from control mice receiving medium. (TIF) [file ppat.1004414.s003.tif]

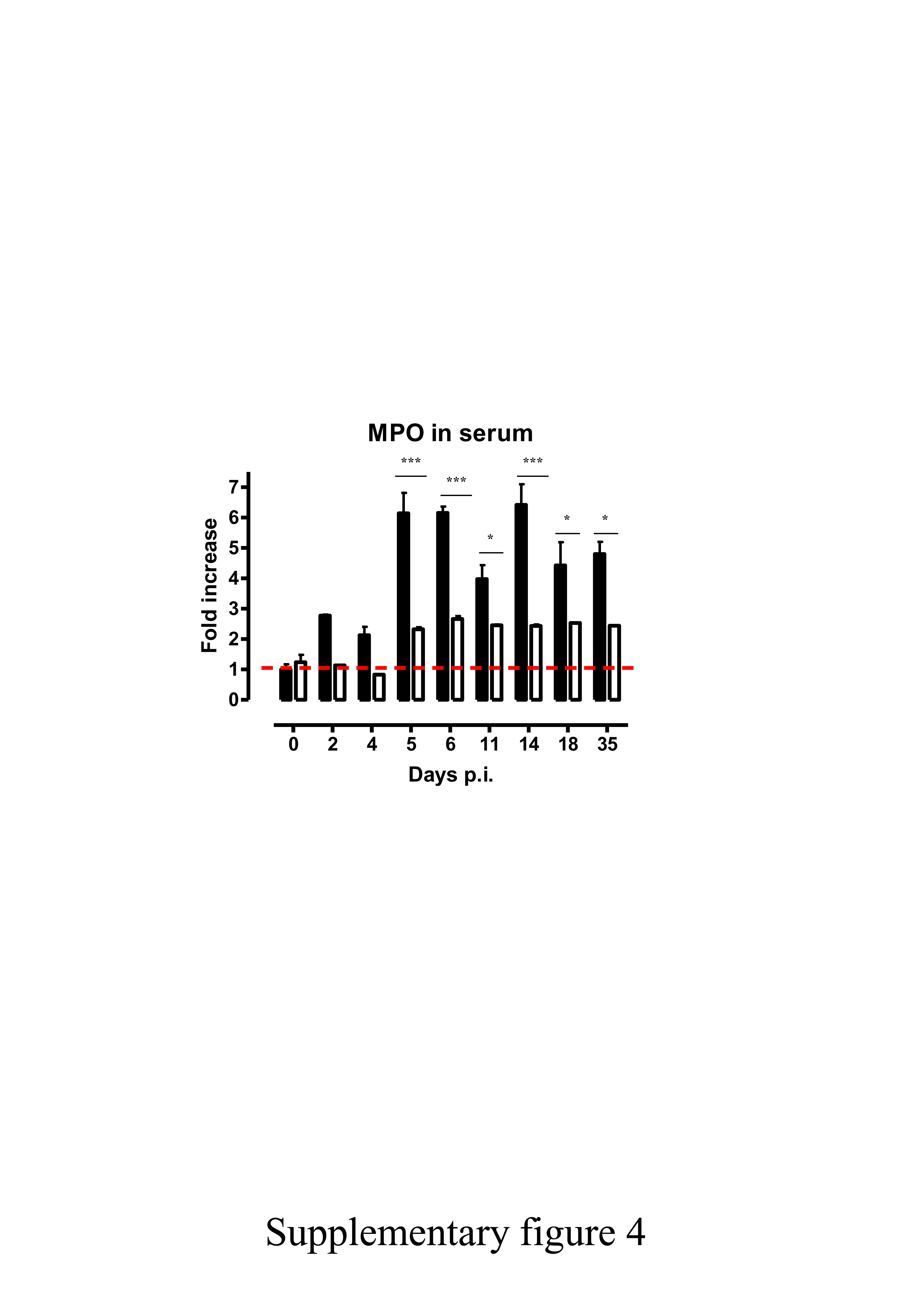

Supplement: Figure S4 — Serum myeloperoxidase activity increases during T. brucei infection. Fold increase in serum myeloperoxidase (MPO) in WT (black box) and Mif −/− (white box) C57Bl/6 mice (compared to levels in non-infected mice, dashed line). Results are representative of at least 2 independent experiments and expressed as mean of 5 individual mice ± SEM. (*: p-values ≤0.05, **: p-values ≤0.01, ***: p-values ≤0.001). (TIF) [file ppat.1004414.s004.tif]

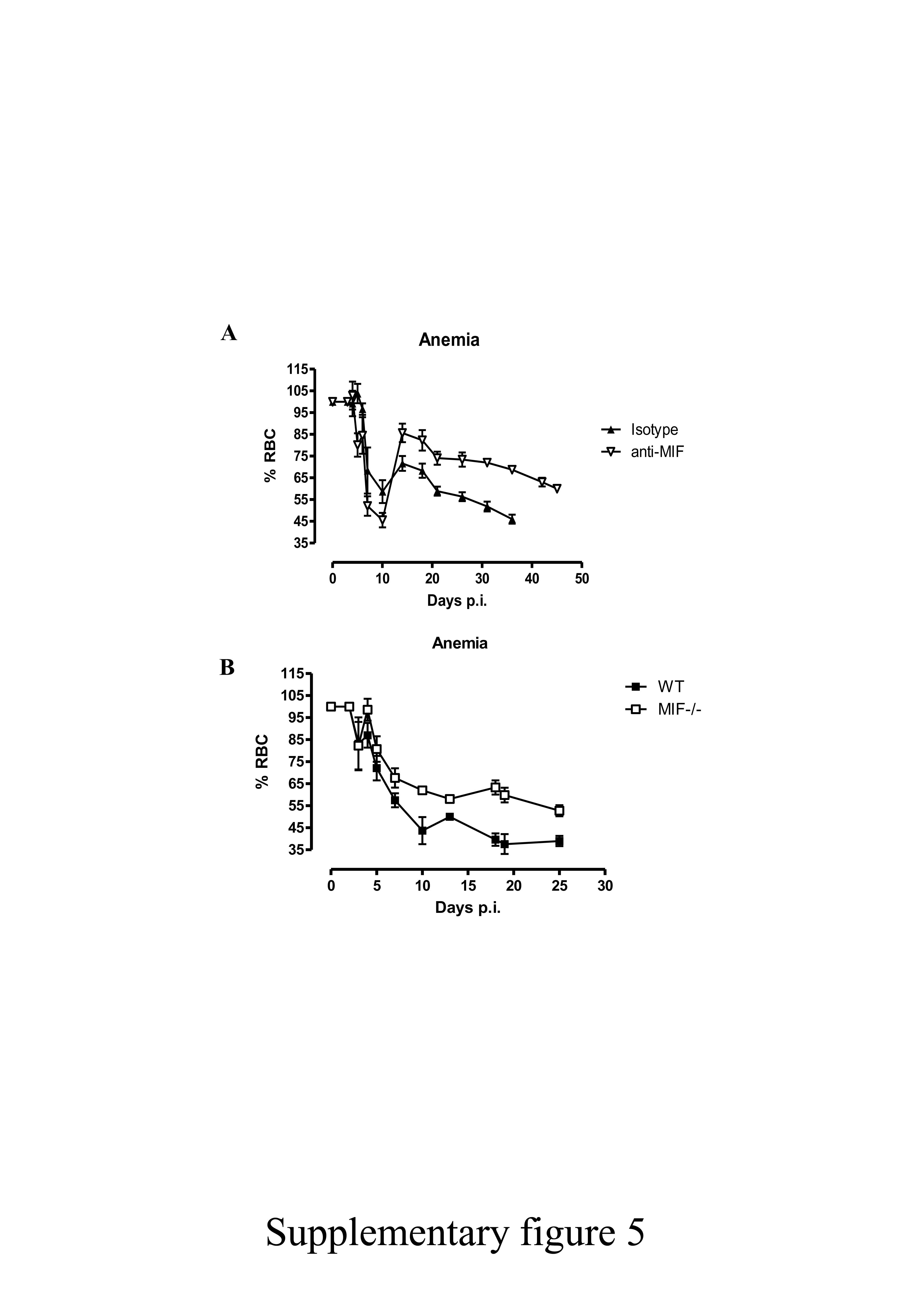

Supplement: Figure S5 — MIF deficiency reduces anemia during anti-MIF treatment and tsetse fly mediated T. brucei infection. (A) Anemia levels following i.p. injection of isotype control (black triangle) and anti-MIF IgG treated (inverted white triangle) WT mice. (B) Anemia development during tsetse fly-based infection (AnTar1) infection in C57Bl/6 (WT) (black box) and Mif −/− (white box) mice. Results are representative of at least 3 independent experiments and expressed as mean of 5 individual mice ± SEM. (TIF) [file ppat.1004414.s005.tif]
