# Supplementary material for: MIF Contributes to Trypanosoma brucei Associated Immunopathogenicity Development
Source: PLoS Pathog. 2014 Sep 25;10(9):e1004414. doi: 10.1371/journal.ppat.1004414 (PMC4177988; doi:10.1371/journal.ppat.1004414)
Supplement: Table S1 — Primers used for RT-QPCR analysis. Table summarizing the gene names and corresponding primer sequences of all primers used in this manuscript. All primers were purchased at Sigma and designed using the Primer-Blast software (NCBI). (DOCX) [file ppat.1004414.s006.docx]

**Table S1: Primers used for RT-QPCR analysis ^1^**

| **Gene** | **Primer sequence** |
| --- | --- |
| *Mif* (Macrophage migration inhibitory factor) | F: 5’-CTTTTAGCGGCACGAACGAT-3’  R: 5’-AAGAACAGCGGTGCAGGTAA-3’ |
| *Hmox-1* (Heme oxygenase 1) | F: 5’-GACACCTGAGGTCAAGCACAG-3’  R: 5’-CCACTGCCACTGTTGCCAAC-3’ |
| *Dmt1* (Divalent metal ion transporter 1) | F: 5’-TCATGGAGGGATTCCTGAAC-3’  R: 5’-TCCTCCAGCCTATTCCATTG-3’ |
| *Fpn1* (Ferroportin-1) | F: 5’-CCAGTCATTGGCTGTGGTTT-3’  R: 5’-AGGTGGGCTCTTGTTCACAT-3 |
| *Fth1* (Ferritin Heavy Chain) | F: 5’-GTCAGCTTAGCTCTCATCAC-3’  R: 5’-ACGTCTATCTGTCTATGTCTTG-3’ |
| *Gata1* (GATA binding protein 1, Erythroid transcription factor) | F: 5’-GCTGACTTTCCCAGTCCTTT-3’  R: 5’-CCGGTTCTGACCATTCATCT-3’ |
| *Epor* (Erythropoietin receptor) | F: 5’-TTTGAGGGTCTCTTCACCAC-3’  R: 5’-GGCAGCAACCACTTATCCAA-3’ |
| *Tal1* (T-cell acute lymphocytic leukemia protein 1) | F: 5’-TAGGGCAAGCAAAGCAAGAG-3’  R: 5’-AGACCATCAGCCAACAGACA-3’ |
| *Maea* (Macrophage erythroblast attacher, *Emp*) | F: 5’- GCATCGAGGACCTTGTGAAT-3’  R: 5’-TCCTGAATCCTGAGGCTGAA-3’ |
| *Gas6* (Growth arrest-specific protein 6) | F: 5’-GATGTCAATGAGTGTGTCCAGA A-3’  R: 5’-GCAAGCGAGAAGCCACTATG-3’ |

^1^ purchased at Sigma, designed using the Primer-Blast software (NCBI)
