# Supplementary material for: MIF Contributes to Trypanosoma brucei Associated Immunopathogenicity Development
Source: PLoS Pathog. 2014 Sep 25;10(9):e1004414. doi: 10.1371/journal.ppat.1004414 (PMC4177988; doi:10.1371/journal.ppat.1004414)
Supplement: Table S2 — Ct values via RT-PRC for the household gene s12 of naïve and infected mice. Overview of the Ct values for the household gene s12 for naïve and T. brucei infected mice at the level of the bone marrow, spleen and liver. Note: Results are representative of 2–3 independent experiments and presented as mean of 3 individual mice ± SD. (DOCX) [file ppat.1004414.s007.docx]

**Table S2: Ct values via RT-PRC for the household gene *s12* of naïve and infected mice**

|  | Bone marrow | Spleen | Liver |
| --- | --- | --- | --- |
| Naïve mice | 18.85 ± 0.78 | 18.5 ± 0.71 | 19.8 ± 0.85 |
| Infected mice | 17.95 ± 0.35 | 18.03 ± 0.59 | 19.4 ± 0.57 |

Note: Results are representative of 2-3 independent experiments and presented as mean of 3 individual mice ± SD
